# SARS-CoV-2 precipitating a stroke in a child? A case report from Tanzania

**DOI:** 10.11604/pamj.2022.42.33.33018

**Published:** 2022-05-12

**Authors:** Rukhsar Shabir Osman, Saliha Shafik Dawood, Sheliza Parvez Thawer, Shivangi Mukesh Mandania, Mudassir Hussein Amirali, Maria Nathaniel Bulimba, Nahida Zahir Walli, Edward Nkingwa Kija, Hussein Karim Manji

**Affiliations:** 1 The Aga Khan Hospital, Dar-es-salaam, Tanzania,; 2 Muhimbili University of Health and Allied Sciences, Dar-es-salaam, Tanzania

**Keywords:** SARS-CoV-2, COVID-19, pediatric stroke, Tanzania, case report

## Abstract

There is scanty data on overall pediatric presentations with COVID-19 in sub-Saharan Africa and none reported related to stroke. Management of acute stroke in children has been challenging due to delays in presentation and difficulties in deducing the exact etiology. This is the first such case of a stroke in a child with COVID-19 infection reported in Tanzania to the best of our knowledge. A six-and-a-half-year-old male child of Asian origin with no history of chronic illness presented to our facility with fever, rash, gastrointestinal symptoms and conjunctivitis. Subsequently, he developed headache, irritability, altered mentation, loss of speech, facial nerve palsy and hemiparesis. He was provisionally diagnosed with bacterial meningitis with a differential diagnosis of viral encephalitis and received standard treatment for the same. On further investigations, magnetic resonance imaging (MRI) of the brain showed ischemic infarct along the territory of left middle cerebral artery and given the history of the child´s exposure to a relative with COVID-19 infection, child underwent a nasopharyngeal swab for polymerase chain reaction testing which was negative but the serum IgG for COVID was positive. Despite the severe presentation initially, early detection and appropriate management resulted in survival, regained speech and motor function. Due to constraints in health care systems in sub-Saharan Africa, it is difficult to exhaust the diagnostics in order to narrow down the list of differentials in a child with stroke. This case is reported to further describe the diverse presentations of COVID-19 particularly in children which has been under-represented especially in sub-Saharan Africa. Attending physicians should have a high index of suspicion for SARS-CoV-2 as the etiology for exposed children presenting with neurological symptoms.

## Introduction

The novel Severe Acute Respiratory Syndrome Coronavirus 2 (SARS-CoV-2) was first reported in the city of Wuhan, China in 2019 (thus called COVID-19) affecting mostly the elderly causing respiratory symptoms ranging from mild, moderate, severe with some leading to respiratory failure and death. Other symptoms including neurological manifestations have also been reported. Children have been mostly asymptomatic while a few develop mild symptoms with rare cases of neurological complications related to SAR-CoV-2 reported in the pediatric population [1].

Although there are reports that children are less likely to develop COVID-19 infection, it is important to monitor the symptoms and the course of the disease carefully. Coronavirus infections are reported to affect the airways in a majority of cases but some pathophysiological mechanisms may be poorly understood as they are known to pass through the epithelium barrier of the nasal cavity and reach the bloodstream or lymph and propagate towards other tissues, including the central nervous system [2]. Children with COVID-19 can present with complications related to different systems including neurological complications. A child may present with symptoms of either a hemorrhagic or ischemic stroke upon presentation to a health care facility. Neurological presentation in pediatric patients with COVID-19 is associated with high mortality rates even in those without comorbidities. According to a study done in Peru, cerebrovascular events occurred in 12.8% of pediatric patients diagnosed with COVID-19 of whom only one had an underlying comorbidity and more than half of them died. Those who survived had significant sequelae [3].

Available data in sub-Saharan Africa regarding stroke associated with COVID-19 infection are limited to the adult population. According to our knowledge, there are no reports of COVID-19 associated with stroke in the pediatric population in sub-Saharan Africa. This is the first pediatric case of COVID-19 and stroke reported in Tanzania. Despite an increased incidence of pediatric stroke, there is often a delay in diagnosis and cases may still remain under or misdiagnosed especially in sub-Saharan Africa where a scarcity of resources prevails. During this era of the pandemic, vigilance by medical professionals about neurological symptoms in children is of utmost importance as they can have long term sequelae which can affect their quality of lives.

Neurological signs in children with COVID-19 is estimated to be 2.4% with most cases being asymptomatic. The symptomatic cases of COVID-19 in children are mostly mild to moderate in severity with few cases of severe presentation and death. In adults, neurological signs were found in at least 84%, which can later lead to other severe COVID-19 complications such as pulmonary thrombosis [4]. Outside Africa, there are a few cases reported of adolescents with COVID-19 with focal cerebral arteriopathy or cardioembolic thrombosis, stupor, meningitis, Lemierre syndrome and intracranial vasculitis. The emphasis from these reports is directed mostly to parents and relatives to take early necessary measures including attending medical facilities when the child shows early symptoms, self-isolation and sanitary measures [5]. In this report, we describe stroke in paediatric population associated with COVID-19 infection, the first reported from Tanzania to the best of our knowledge.

## Patient and observation

**Figure 1:**
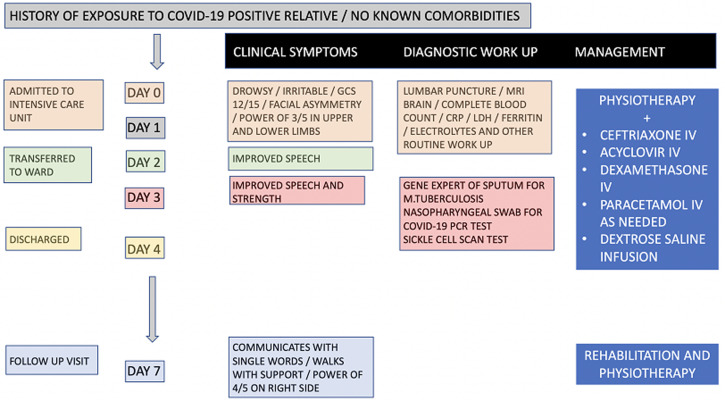
timeline of events

A six-and-a-half-year-old fully vaccinated Asian boy presented to the accident and emergency department with 5-day history of high-grade fever associated with non-projectile vomiting, loose stools, abdominal pain, generalized rashes and conjunctivitis without discharge. He also had history of reduced oral intake and urine frequency. However, he had no history of cough, joint or limb pain, sore throat or rhinorrhea. He was treated for a suspected bacterial infection at another health facility, with co-amoxiclav and paracetamol then cefixime, ibuprofen and desloratidine. As the illness progressed, he became extremely weak associated with headache and on the day of presentation at our facility he was irritable, had lost speech with mouth deviation towards the left side. He had no reported convulsions, visual disturbance, difficulty swallowing, drooling, difficulty breathing or incontinence. A week prior to his illness, he was exposed to a relative suffering from COVID-19 infection. The sequence of events is as stipulated in the timeline of the patient (Figure 1). His past medical and birth history was otherwise unremarkable with normal growth and developmental milestones; no family history of sudden death, seizures, malignancy, cardiac or sickle cell disease.

**Clinical findings:** on examination, he was found to be drowsy and irritable with altered mental status and a Glasgow coma scale of 12/15 associated with facial asymmetry and non-purulent conjunctivitis. Vital signs were normal on presentation. On neurological examination, he was found to have bilaterally equal and reacting pupils, mouth deviation to the left, with loss of brow wrinkle and nasolabial fold on the right, drooping of the mouth corner on the right, stiff neck with negative Kernig and Brudzinski signs, right sided hemiparesis with a power of 3/5 on both right upper and lower limbs, positive right ankle clonus and hyperreflexia of deep tendon reflexes. Cardiovascular, respiratory and abdominal examination findings were unremarkable. A differential diagnosis of meningitis and encephalitis was made.

**Diagnostic assessment:** lumbar puncture was done and analysis revealed cerebrospinal fluid (CSF) was blood stained, no white blood cells (normal: 0-5 cells/mm^3^) and numerous red blood cells (normal: nil), protein was 52.87mg/dl (normal: 15-45 mg/dl), glucose was 4.22 mmol/L (normal: 2/3 of blood sugar which is around 6.8 mmol/l), no organism growth on culture and no pathogen deoxyribonucleic acid (DNA) or ribonucleic acid (RNA) detected on polymerase chain reaction (PCR). Complete blood count showed white blood cell (WBC) of 26.23 x 10^9^/l (range: 4.0 -14.5 x 10^9^/l) with absolute neutrophils 22.90 x 10^9^/l (range: 1.78 - 5.38 x 10 ^9^/l), lymphocytes 1.59 x 10 ^9^/l (range: 0.8-4.0 x 10 ^9^/l), hemoglobin 11.4g/dl (range: 10.8-12.8 g/dl), platelets 245 x 10^9^/l (range: 150-450 x10 ^9^/l); blood culture was sterile. C-reactive protein (CRP) was 250.56 mg/l (range: 0.5 -5 mg/l), liver and renal functions were normal, electrolytes showed a mild hyponatremia of 132 mmol/l (normal 135-145 mmol/l), serum ferritin, lactate dehydrogenase and lactate was elevated: 517.87 µg/l (range: 30-400 µg/l), 247 IU/l (range: 135-225 IU/L) and 3.48 mmol/l (range: 0.5-2.2 mmol/l) respectively (Table 1).

**Table 1:** laboratory Investigations

Date	Investigation	Results
On admission	CSF	No WBC, numerous RBC, protein 52.87mg/dl, no organism growth, no pathogen DNA or RNA detected
	WBC	26.23 x 10^9^/l
	Absolute neutrophils	22.90 x 10^9^/l
	Lymphocytes	1.59 x 10^9^/l
	Hemoglobin	11.4g/dl
	Platelets	245x 10^9^/l
	Blood culture	Sterile
	CRP	250.56mg/l
	Sodium	132 mmol/l
	Ferritin	517.87μg/l
	LDH	247 IU/l
	Lactate	3.48mmol/l
Day 1	Gene x-pert on the sputum	Negative for mycobacterium tuberculosis
	Nasopharyngeal swab for COVID-19 PCR	Negative
	Sickle cell scan test	HbAA
Day 4	WBC	25.37 x 10^9^/l
	CRP	60.96mg/l.
Day 6	WBC	24.23 x 10^9^/l
	CRP	20.45mg/L
	COVID-19 antibody test	IgG positive

CSF: cerebrospinal fluid; WBC: white blood cells; CRP: C-reactive protein; LDH: lactate dehydrogenase; PCR: polymerase chain reaction; RBC: red blood cells; DNA: deoxyribonucleic acid; RNA: ribonucleic acid

**Figure 2:**
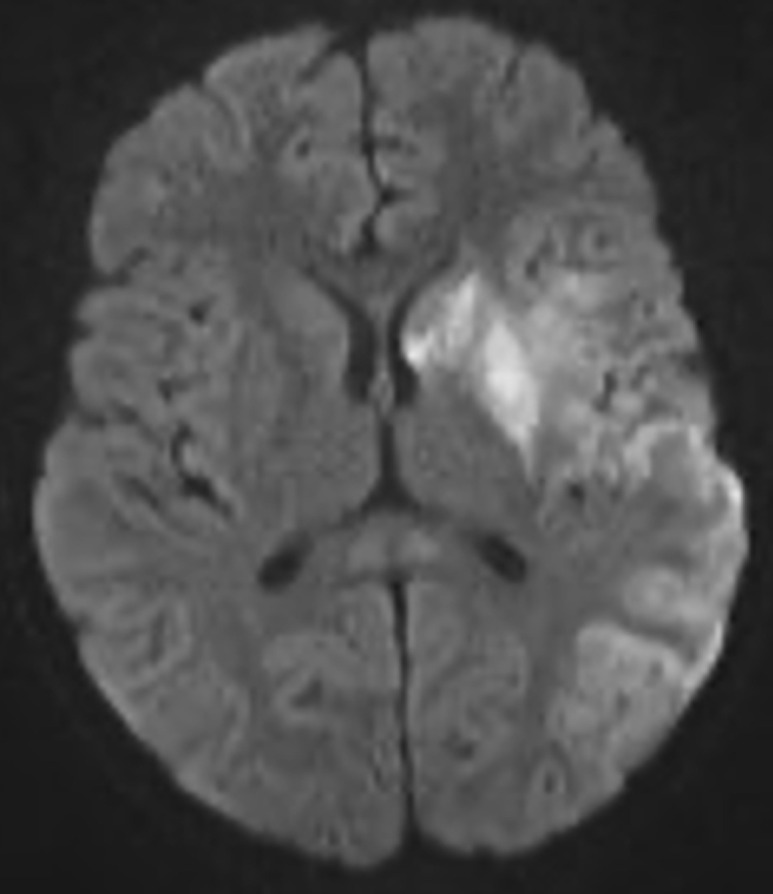
diffusion axial slice of MRI brain showing areas of restriction on which are involving caudate nucleus, putamen, insular cortex and parietal lobe

**Figure 3:**
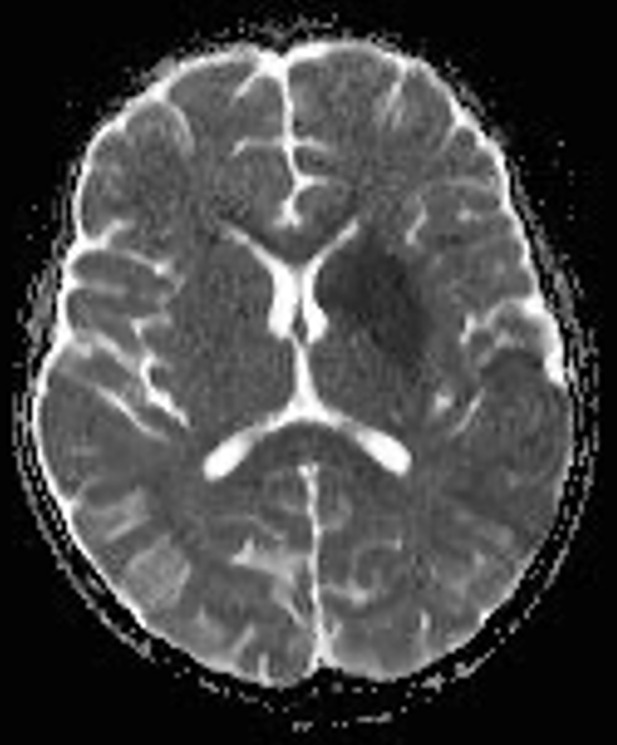
ADC axial slice of MRI brain showing areas of restriction on diffusion weighted images are evident involving caudate nucleus, putamen, insular cortex and parietal lobe

A brain MRI was done which revealed acute ischemic infarct along the territory of left middle cerebral artery involving caudate nucleus, putamen, insular cortex, and left parietal lobe (Figure 2, Figure 3). Gene X-pert on the sputum was negative for mycobacterium tuberculosis, nasopharyngeal swab for COVID-19 PCR was also negative and sickle cell scan test showed HbA. Also, vascular imaging and echocardiography were not done.

**Therapeutic intervention:** he was admitted to the intensive care unit (ICU) with a diagnosis of a cerebrovascular infarction with COVID-19 infection with differentials of meningitis still being covered for and started on intravenous (IV) ceftriaxone at 100 mg/kg/day and IV acyclovir at 10 mg/kg 8 hourly to cover for both bacterial and viral meningitis, IV dexamethasone 0.6 mg/kg single dose on the first day before antibiotics administration followed by 0.15 mg/kg every 6 hourly for the following 3 days and IV paracetamol at 15 mg/kg every 8 hourly. IV normal saline at 10 ml/kg was administered with maintenance on IV dextrose saline at 65 ml/hour. Rehabilitation with physiotherapy and occupational therapy was also initiated.

**Follow-up and outcomes:** rehabilitation and medication was continued with further improvement in his speech and strength, and he was discharged on the fourth day post admission on IV ceftriaxone and IV acyclovir to complete 7 and 14 days respectively. Repeat tests prior to discharge showed WBC: 25.37 x 10^9^/l and CRP 60.96 mg/l. During the hospital stay, no adverse or unanticipated events occurred in the child and he improved clinically with time. Treatment adherence and tolerability was assessed by a close follow-up. On follow-up, 2 days later, he was able to communicate using single words, walk with support with right leg still dragging. On examination right side power improved to 4/5 with brisk deep tendon reflexes and sustained clonus. Repeat CRP: 20.45 mg/L, WBC: 24.23 x 10^9^/l; COVID-19 antibody test: IgG positive. He was seen again 2 weeks later in the outpatient setting and was able to communicate in sentences, ambulant with dragging of right leg and power of 4/5 in upper and lower limbs of right side with brisk deep tendon reflexes and was advised to continue with physiotherapy and occupational therapy.

**Informed consent:** informed consent was obtained from the parents given the patient was a minor.

## Discussion

Neurologic presentation in pediatric patients with COVID-19 has been reported to be a rare but nevertheless an important prognostic factor. One possible mechanism associating COVID-19 to ischemic stroke is that the virus potentiates a prothrombotic and proinflammatory state via endothelial cell disruption and clotting cascade activation [6]. It has been suggested that the relationship between COVID-19 and stroke involves large artery occlusion, something quite exclusive in COVID-19 associated stroke and exhibiting a characteristic pattern of infarcts in multiple arterial territories. The authors also conveyed that the majority of strokes are ischemic with only approximately 10% being hemorrhagic [7].

Acute ischemic stroke has been reported in adults but there are very few reported cases in the pediatric population in the west and none so far in sub-Saharan Africa. This ignites the need to conduct studies and establish a high index of suspicion in this population. According to a recent study done in Peru [3], 23.4% of patients admitted with COVID-19 presented with neurologic complications. In another study where surveys were done in 61 international sites with pediatric stroke expertise and this study revealed that SARS-CoV-2 was detected in 4.6% of pediatric patients with features of ischemic stroke who were tested for the virus [8]. This emphasized the need for COVID-19 testing to be considered in all pediatric cases presenting with neurologic manifestations with a high index of suspicion to allow health care workers manage them effectively without causing any diagnostic delay. Our case described above presented to us after a prolonged duration of persistent fevers accompanied with an erythematous rash, non-purulent conjunctivitis, non-projectile vomiting and loose stools. These features are in line with classical signs and symptoms of COVID-19 as suggested by the literature and from a clinical experience perspective.

The child also had aphasia, loss of facial symmetry, neck stiffness, mouth deviation to the left side and right sided hemiparesis which were noted on presentation of the child to the hospital. In relatable studies, there was a report of a 17-month-old with positive antibodies for COVID-19 who presented with an acute onset of right hemiparesis and a 10-year-old girl who tested positive on PCR for COVID-19 who had presented with facial distortion, severe headaches and left hemiparesis. Both cases were preceded with a history of fevers and gastrointestinal manifestations [1,9]. While multiple differentials were considered, COVID-19 was one among them especially given the history of exposure of the child to a relative who had been diagnosed with the same.

Coupled with the hematologic parameters revealing leukocytosis and significantly elevated infectious markers including C-reactive protein, lactate dehydrogenase and serum ferritin; the clinical features and early laboratory findings led us to an initial diagnosis of acute bacterial meningitis. On further investigation, we acquired a negative meningitis panel, absence of white counts in the cerebrospinal fluid analysis and no growth on cultures leading us to rule out both the diagnoses. Magnetic resonance imaging done revealed an acute ischemic infarct along the territory of left middle cerebral artery involving caudate nucleus, putamen, insular cortex, and left parietal lobe. In the 17-month-old mentioned earlier, magnetic resonance imaging of the brain revealed a left pontine infarct [9]. Another study conducted in Iran on a 12-year-old boy with COVID-19 reported focal cerebral arteriopathy on MRI which happens to be a common cause of pediatric stroke [10]. With the era of the pandemic, the rates of strokes have increased [4] and the cerebrovascular system has been implicated to also be affected by this virus. The proposed mechanisms involve proinflammatory and hypercoagulable states which cause thrombotic events resulting in this presentation. This report suggests an association between SARS-CoV-2 and stroke in children and contributes to the pool of knowledge to encourage clinicians to maintain a high index of suspicion in children with this presentation for early diagnosis and management accordingly.

Sickle cell disease is likely the most common underlying risk factor for pediatric stroke worldwide and given the highest prevalence of sickle cell disease in sub-Saharan Africa, sickle cell screening was done for our patient and was negative [11]. This was identified as one of the strengths in this case. Viral infections can be a trigger for cardioembolic, arteriopathy or idiopathic stroke in children. Some limitations in our study included that vascular imaging was not done to rule out cerebral arteriopathy. Secondly, echocardiography was important but was not done to rule out congenital heart diseases since clinical assessment and laboratory parameters were highly suggestive of infectious origin of ischemic stroke in our patient. Also, hematological abnormalities such as deficiency of protein C and S are rare and its evaluation are not routinely done in our setting.

## Conclusion

Due to the few reported cases of COVID-19 and pediatric strokes, concerns may be raised on the numbers of missed cases and diagnostic delays especially in low resource settings. It is recommended that a diagnosis of COVID-19 be entertained for any febrile illness lasting longer than 3 days and presenting with mucocutaneous findings even in the absence of respiratory symptoms. Any child presenting with persistent fevers should promptly be referred to and evaluated at a tertiary center of care to curb the problem of under diagnosis and misdiagnosis of this condition.
